# Nanoswimmers Based on Capped Janus Nanospheres

**DOI:** 10.3390/ma15134442

**Published:** 2022-06-24

**Authors:** Petteri Piskunen, Martina Huusela, Veikko Linko

**Affiliations:** 1 Biohybrid Materials, Department of Bioproducts and Biosystems, Aalto University, P.O. Box 16100, 00076 Aalto, Finland; petteri.piskunen@aalto.fi (P.P.); martina.huusela@aalto.fi (M.H.); 2 LIBER Center of Excellence, Aalto University, P.O. Box 16100, 00076 Aalto, Finland

**Keywords:** nanoswimmers, nanospheres, Janus particles, biohybrids, targeted delivery, nanofabrication, biomedicine

## Abstract

Nanoswimmers are synthetic nanoscale objects that convert the available surrounding free energy to a directed motion. For example, bacteria with various flagella types serve as textbook examples of the minuscule swimmers found in nature. Along these lines, a plethora of artificial hybrid and non-hybrid nanoswimmers have been introduced, and they could find many uses, e.g., for targeted drug delivery systems (TDDSs) and controlled drug treatments. Here, we discuss a certain class of nanoparticles, i.e., functional, capped Janus nanospheres that can be employed as nanoswimmers, their subclasses and properties, as well as their various implementations. A brief outlook is given on different fabrication and synthesis methods, as well as on the diverse compositions used to prepare nanoswimmers, with a focus on the particle types and materials suitable for biomedical applications. Several recent studies have shown remarkable success in achieving temporally and spatially controlled drug delivery in vitro using Janus-particle-based TDDSs. We believe that this review will serve as a concise introductory synopsis for the interested readers. Therefore, we hope that it will deepen the general understanding of nanoparticle behavior in biological matrices.

## 1. Introduction

Nanotechnology provides new material properties and solutions for several research fields, including medicine, material science and biochemistry [1]. One important class of revolutionary new materials comprises various nanoparticles, which give rise to a plethora of applications [2]. There exist numerous different types of particles and their advanced assemblies [3], each with their own specific implementation across the various fields of science and engineering. One of the most exiting branches of these nanoscopic particles is the various kinds of micro- and nanomotors, or “swimmers” [4,5], that can be created with them.

Biological micro- and nanoscale motors, such as kinesin or bacteria with flagella [6], have been intensively studied to understand smaller-scale kinetics. Therein, they have served as inspiration for researchers aiming to develop remotely controlled particles. Thus, mobile nanoparticle units, nanoswimmers (NSs), can be defined as synthetic versions of biological swimmers. These synthetic tools can be used as machines to probe, sense and traffic cargo in biological systems [7,8,9] and for modeling the intricate real-world functions of biological swimmers and other small-particle systems.

Similarly to biological swimmers, synthetic swimmers are subject to the viscous and thermal forces that dominate in the microscopic world [4,8,10]. Due to the small size of NSs, their inertia is insignificant compared to the viscous forces of their surroundings, and thus their motion demands constant power. This makes nanoscale motors different from larger ones and brings challenges to their development [6,11]. Such small particles are also affected by thermal forces and Brownian motion [12], which causes the particles to vibrate and collide, resulting in random movement [8]. Moreover, as the particle size decreases further, the relative Brownian forces increase. This presents challenges in the design [13] and characterization [14] of controllable mobile swimmers.

Although the morphologies of these nanoswimmers can be as varied as their uses, the two-faced Janus particles (JPs) [15,16,17] and the simple capped Janus nanospheres (CJNs) [5] have seen much use as the core elements. In particular, the capped nanospheres can be manufactured quite straightforwardly from biocompatible materials, and, thanks to their two-faced nature, they can be decorated with modifications selectively to add to their functionality. This has allowed JPs to form the basis for both biohybrid and non-hybrid nanoswimmers with different propulsion mechanisms and uses, leading to potential applications in targeted drug delivery systems (TDDSs) [7], microsurgery [18] and water remediation [19], to name a few. Recently, swimmers built using fully synthetic biomolecular components, such as DNA nanostructures [20,21], have also appeared.

**Figure 1:**
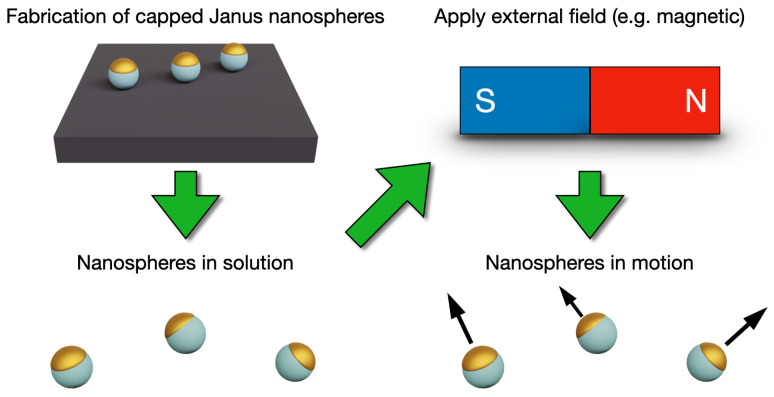
Artistic rendering of the theme of this review. Capped Janus nanospheres can be fabricated, for example, on a supporting substrate and further resuspended in solution. Upon the introduction of a chosen external field or fuel, the nanospheres elicit a directional movement.

This review discusses the properties and fabrication of capped Janus nanospheres and their use in nanoswimmer systems (Figure 1). As the literature on Janus nanoswimmers is vast, we focus on summarizing the main features using prominent examples, rather than giving a thorough list of all works. We also discuss select recent biohybrid swimmers that have employed CJNs as their foundation, as well as the new biohybrid swimmers based on synthetic DNA nanostructures.

## 2. Nanoswimmers

Nanoswimmers have several means to move around in a liquid environment. The spatial and temporal movement of NSs can be controlled in different ways depending on the propulsion styles of the particles. Similarly to natural swimmers, some synthetic NSs convert the energy from their surroundings into movement via chemical reactions [22], heat [23,24], light [25] or magnetic fields [26] depending on the properties of the particles. Based on the exact mechanism, NSs can be divided into groups of autonomous and non-autonomous swimmers. Autonomous swimmers possess an energy source in the particle itself, whereas non-autonomous NSs require external stimuli (a magnetic field, etc.) to actuate the movement [11].

The movement of autonomous swimmers is usually based on chemical reactions. These kinds of motor-embedded particles move using the energy from either fuels or enzymatic reactions. The fuels and enzymes are located asymmetrically in a specific region of the particle to induce a gradient-based effect [27], self-electrophoresis [5] or localized chemical reactions [28,29] and bubble formation [30]. Fuels, however, often show toxicity, motivating the development of other self-propulsion mechanisms. Thus, autonomous NSs have been developed. For instance, electrical fields have been used to move particles, but this mechanism is generally suitable only for non-biological systems [8]. Conversely, magnetic fields can be used for biological purposes without the risk of toxicity and are very low-risk when used in a low-strength and low-frequency regime. Magnetic fields can additionally penetrate biological tissues without disturbance and enable a remote way of controlling particle movement. Recently, acoustic waves have also been investigated as an energy source for NSs [31] and swimmers with flagellum have been shown to react to both traveling and standing waves [32]. NSs have also been developed for several purposes ranging from radiotherapy tools [33] to contrasting agents [34] and diagnostic devices [35].

Nanoswimmers can be manufactured from several materials, including polymers, metals, carbon and other inorganic compounds. The choice of materials affects the application and properties of the particles. Metals, such as gold, exhibit several advantageous properties (electronic, magnetic, optical and catalytical) that can be useful in functionalizing nanoparticles with other materials (polymers, biomolecules, etc.) [36]. Therefore, gold has been widely used in biological NS applications. In general, to use NSs in the biomedical fields, different fabrication methods should yield particles of uniform quality, since even small fluctuations in the particle shape, size and surface properties may result in changes in particle behavior [37].

## 3. Classification and Fabrication of Capped Janus Particles

Nanoparticles (NPs) are small objects with at least one of their dimensions in 1–100 nm size. They often exhibit dissimilar physical properties than larger objects of the same matter, caused by the exponential growth of relative surface area, as well as the emphasis of interactions between molecules and atoms, as size decreases [2]. The size of NPs enables them to penetrate biological barriers that would not otherwise be penetrable, and they possess higher solubility and reactivity than larger particles. NPs can be of various shapes, including rod-like, spherical and tetragonal, among others [2], and they can comprise a variety of materials and their combinations. Furthermore, the surface properties of NPs may be altered by the addition of functionalization groups and they can be loaded with dyes and drug molecules.

Janus particles, named after the two-faced Roman god Janus, are particles that have two distinct and asymmetric sides, which result in asymmetric chemical and physical properties [8,38]. The term Janus particle first appeared in the 1962 novel “The Mouse on the Moon”, where it depicted a space travel device using wine as a propellant [39], analogous to the JP nanoswimmers in solution discussed here.

JPs generally possess two distinct surface areas, which serves as a foundation for different functions [1,17]. For example, surface modifications, such as thiol groups [2], can be selectively conjugated to the particles to create localized patches for further functionalizations. These characteristics enable the assembly of multifunctional and multicomponent swimmers that are capable of cargo transport [40], self-propulsion [30], sensing [35] and dynamic behavior [35]. This possibility for modular property combinations is a major reason for researching JPs. Thus, they have been widely investigated in previous years due to their diverse properties and complex behavior that enable several simultaneous functions in one particle, such as the self-generated propulsion and magnetic guidance that are discussed later in this article. Owing to this versatility, JPs often form the foundation especially for nanomotor and drug delivery applications.

### 3.1. Capped Janus Nanoparticles

As a conceptually simple variant of JPs, CJNs feature a localized coating or asymmetric ligand binding on their surfaces, i.e., a “cap” of deposited material. This partial coating can be used as a localized attachment surface for other components to bestow them with more interesting abilities such as movement and targeting. These kinds of surface modifications enable, for example, the attachment of biological components such as DNA strands [41] to inorganic noble metal and metal-oxide swimmers [1,2].

### 3.2. Synthesis of Capped Janus Nanospheres

**Figure 2:**
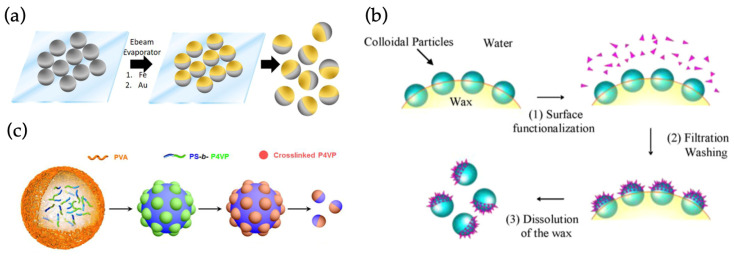
The three main mechanisms of Janus particle fabrication, illustrated with selected examples. (**a**) Masking: electron beam evaporation or sputtering can be used to deposit material on surface-attached spheres. (**b**) Phase separation: phase boundaries (here, water–wax interface) can be used to direct assembly. (**c**) Self-assembly: For example, copolymers can be selectively cross-linked and then disassembled to yield Janus spheres. (**a**) reproduced from Ref. [45]. Copyright 2017 American Chemical Society. (**b**) reproduced from Ref. [46]. Copyright 2009 Elsevier. (**c**) reproduced from Ref. [47]. Copyright 2015 American Chemical Society.

JPs can be manufactured by several different techniques from multiple materials. The materials are chosen to serve a certain function and they can comprise several different mixtures of substances, such as carbon–silica or polymer–metallic oxide [38]. Some JPs are manufactured by using a core material, such as silica, which is then partly or fully covered with another material [42]. In biomedical applications, materials such as mesoporous silica or gold have been widely adopted due to their biocompatibility. Mesoporous silica is effective for drug loading, is biocompatible and can be combined with several other key materials [43]. JP manufacturing techniques include, for example, masking, phase separation, self-assembly, the Pickering emulsion technique, free-radical polymerization, polymer grafting and the usage of microfluidic channels [1,15,17,42,44]. However, the primary techniques can be generally divided into three categories, masking, phase separation and self-assembly, which are illustrated in Figure 2.

Masking (Figure 2a) is a straightforward way to obtain two different sides to a particle by simply covering the other side with another substance. Usually, masking is performed by sinking or trapping the other side of the particle to another substance, while the other hemisphere is covered via adsorption or deposition. Material-wise masking is extremely adaptable, as it allows the creation of metal, semiconductor and even alloy coatings and multilayers. Thus, the method is considered to be an easy route for making a variety of JPs, but it has some limitations with scaling and further functionalization of the surfaces. Masking is still considered to be the most flexible manufacturing method, which can be applied to most particle materials and recently also cells [48]. Phase separation (Figure 2b) can be used to achieve more intricate NPs than the masking method. JPs, such as quantum dots, nanocrystals and variations of different heterodimers, are often fabricated via phase separation. The technique is based on the separation of two different compounds in a mixture, which enables the partial modification of particles at the phase boundaries. This method can be used to manufacture particles composed of polymers and inorganic materials or a mixture of both. Benefits of this include creating intricate functionalizations on the surface. The third manufacturing method is self-assembly (Figure 2c), which is used to prepare especially polymeric JPs. This technique rests upon the characteristics of the monomers involved, which leads to the ordered assembly of designer structures [1].

## 4. Capped Janus Particles as Nanoswimmers

### 4.1. Non-Hybrid Swimmers Based on Capped Janus Nanospheres

**Figure 3:**
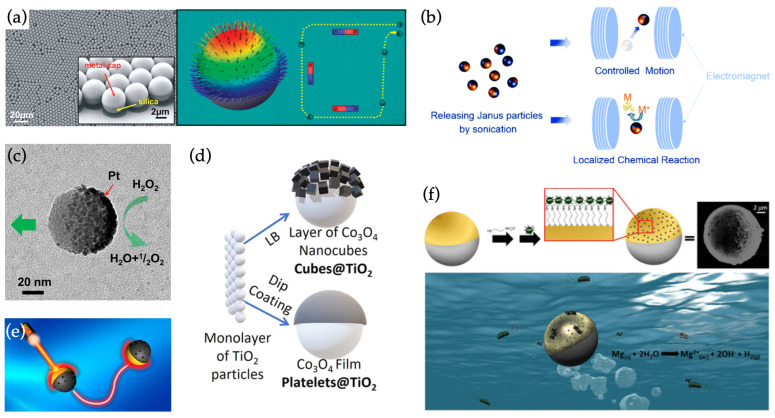
Non-hybrid swimmers made from Janus spheres. (**a**) Left: a metal cap on silica beads induces a magnetic response. Right: magnetic moments in the metal cap at remanence after magnetic saturation. (**b**) Partially coating a particle with magnetostrictive and piezoelectric layers enables dual functions in controlled motion and localized chemical reactions. (**c**) A platinum cap on mesoporous silica enables catalysis-based fuel-free propulsion of a drug-loaded particle. (**d**) Morphological differences in coating affect the swimming behavior of particles. (**e**) Photothermally driven Au-capped silica spheres respond at near-infrared light wavelengths close to the surface plasmon resonance of the coating. (**f**) Mg nanospheres half-coated with a Fe/Au bilayer and topped with bactericidal Ag nanoparticles form multifunctional swimmers for water remediation. (**a**) reproduced from Ref. [49]. Copyright 2012 American Chemical Society. (**b**) reproduced from Ref. [50]. Copyright 2016 Royal Society of Chemistry. (**c**) reproduced from Ref. [47]. Copyright 2015 American Chemical Society. (**d**) reproduced from Ref. [51]. Copyright 2021 American Chemical Society. (**e**) reproduced from Ref. [52]. Copyright 2016 American Chemical Society. (**f**) reproduced from Ref. [45]. Copyright 2017 American Chemical Society.

Even without any further modifications, the partial coating itself can bestow the Janus particles with relatively complex functionality. For example, a self-assembled silica particle monolayer can be covered by a [Co/Pt]${}_{5}$ multilayer stack and characterized using in-plane and out-of-plane magnetic fields (Figure 3a) [49]. In addition, similar particles can be coated with magnetically responsive alloys (a CoFe${}_{2}$O${}_{4}$–BaTiO${}_{3}$ bilayer composite) via sputtering (Figure 3b). There, the spheres firstly exhibit alignment and propulsion, dictated by a rotating magnetic field [50]. Then, owing to the interaction between the magnetostrictive CoFe${}_{2}$O${}_{4}$ and the piezoelectric BaTiO${}_{3}$ layers upon exposure to the magnetic fields, electrical charges could be generated on the particle surface. Thus, in addition to providing the magnetically driven movement, the alloy coatings enable the particles to induce electrochemical reactions, such as the reduction of metal ions, on demand.

In a similar example, Ma et al. [53] fabricated catalytically active nanoswimmers by coating mesoporous silica nanoparticles of various sizes below 100 nm with a very thin (2 nm) platinum layer by using electron beam evaporation (Figure 3c). The platinum coating functioned as a catalytic site for H${}_{2}$O${}_{2}$ breakdown, propelling the particle in the opposite direction from the reaction, even in the presence of low (<3 wt%) concentrations of the H${}_{2}$O${}_{2}$ fuel. The particles could be employed in drug delivery by loading the cavities inside the mesoporous silica with cargo, as demonstrated by transporting Rhodamine B fluorescent dye in vitro. However, the drug release from the particles in this work was constant, as the system contained no mechanism for triggering the release.

These catalysis-based swimmers can also be operated with light in a fuel-free manner. Dong et al. [54] half-coated TiO${}_{2}$ spheres with a Au film to create a similar but photocatalytic swimmer operable at the ultraviolet range. The Au-TiO${}_{2}$ system was driven self-electrophoretically via hydrogen generation induced by light [55] and could be accelerated and decelerated by modulating the intensity of irradiation. Additionally, the swimming speed could be also significantly accelerated with the addition of even very low concentrations (0.1%) of H${}_{2}$O${}_{2}$ fuel.

The swimming mechanisms of these types of TiO${}_{2}$-based swimmers were later investigated by Wang et al. [56] by studying photochemically active Cu-capped TiO${}_{2}$ spheres. The swimmers showed versatile motion depending on the solution and illumination conditions. The particles could be driven in pure water with UV light, but in the presence of H${}_{2}$O${}_{2}$ fuel, they could be activated by both UV and visible light wavelengths. Additionally, as they had both active and passive particles (Au-capped TiO${}_{2}$ that was inactive in their experimental conditions) in their system, it was observed that the active particles created aggregates by shuttling and packing the passive particles in the solution. The packing depended on the motion of the swimmers and was different in the three studied movement conditions. This laid the groundwork for later studies in the propulsion-driven directed assembly in colloidal systems with mixed active and passive particles [57,58,59].

Maric et al. [60] then studied the effects of different coating materials on swimming behavior systematically by fabricating a set of photoresponsive fuel-free TiO${}_{2}$ spheres with partial Pt, Cu, Fe, Ag and Au coatings and analyzing both their structures and behaviors. They concluded that the swimming velocity was a synergistic combination of both the chemical potential differences and catalytic effects of the coating materials in respect to the water splitting reaction that drove the swimmers. The Pt-coated particles exhibited the fastest swimming speeds in their experiments.

By carefully choosing the composition of the two JP surfaces, the photoresponsive properties of both sides can be even exploited individually for more complex control, as was shown by Vutukuri et al. [61]. They showed that Au-capped TiO${}_{2}$ swimmers could be driven with UV light, as in the previous studies, but they also demonstrated that the propulsion direction could be reversed under illumination with green light, which causes H${}_{2}$O${}_{2}$ fuel breakdown to be catalyzed on the Au side, instead of the TiO${}_{2}$. Thus, the particles could be driven in opposite swimming directions by merely changing the illumination wavelength.

Beyond simple material choices, also the morphology and preparation method of the partial coating on the JP will affect their behavior. To illustrate this, Wittman et al. [51] prepared two TiO${}_{2}$ micromotors with morphologically different Co${}_{3}$O${}_{2}$ coatings consisting of either nanocubes or platelets, respectively (Figure 3d). Due to the preparation method of the nanocubes (a Langmuir-Blodgett technique), the Co${}_{3}$O${}_{2}$ cubes were electrically isolated from the TiO${}_{2}$ cores and no photocatalytic propulsion in UV illumination was observed. Conversely, the dip-coated Co${}_{3}$O${}_{2}$ platelets had much better electrical contact with the cores, which did not impede charge transfer or the photocatalytic mechanism between the Co${}_{3}$O${}_{2}$ and TiO${}_{2}$ halves.

It should additionally be noted that fuel-free photoactive swimmers can be made from polymer compounds as well, instead of the common TiO${}_{2}$. Zhou et al. [62] prepared photochemically driven poly(methyl methacrylate) (PMMA) CJNs by half-coating them with a Ag layer through physical vapor evaporation and then converting the Ag into AgCl through incubation in a FeCl${}_{3}$ solution. The PMMA/AgCl particle could then be propelled upon irradiation with UV light or powerful visible light. The authors reasoned that this was a consequence of AgCl decomposition and an electric field gradient between the PMMA core and AgCl layer.

As an alternative to photochemical effects, nanoparticles can also be driven by photothermal effects. By coating the silica particles with a Au cap, the particles can respond to near-infrared light with a photothermal effect (Figure 3e) [52]. The thermal gradient caused by uneven heating between the cap and core particle enhanced the diffusion of the particles upon irradiation at a light wavelength (∼800 nm) near the surface plasmon resonance of the Au film. Similarly to the photocatalytic swimmers, this fuel-free propulsion of the particles could be toggled by switching the irradiating near-infrared laser on and off. Furthermore, by adjusting the power of the laser, the swimming speed of the particles could also be controlled.

In addition to light, thermophoretic effects can also be exploited with magnetic fields. By half-coating SiO${}_{2}$ particles with a permalloy (Fe${}_{1}9$Ni${}_{8}1$) film, the metal cap could be heated when the particles were exposed to an alternating magnetic field, inducing slow propulsion. A separate DC magnetic field could then be used to simultaneously orient the particles in a specified direction [24].

Beyond drug carriers, partially coated nanospheres have also found uses as multifunctional water remediation tools [19], as demonstrated in the work by Vilela et al. (Figure 3f) [45]. A Mg microsphere was partially coated with magnetically responsive Fe, followed by a Au cap (see also Figure 2a). The exposed Mg part of the sphere provided propulsion to the particle, while the Fe enabled magnetic guidance and recovery of the swimmers. The Au half-shell was used for decorating the spheres with Ag nanoparticles, endowing the assemblies with bactericidal properties (adhesion to bacteria and release of Ag${}^{+}$-ions). The bactericidal properties enabled the assemblies to function as water purifying tools. Owing to their adhesive and magnetic properties, the swimmers could also be collected to simultaneously reclaim the swimmers and remove the bacterial contaminants from water samples.

Similarly, Wang et al. [57] designed a photocatalytically driven TiO${}_{2}$ swimmer with a partial Ni and Au coating for water remediation. Drawing inspiration from the previously displayed interactions between active and passive colloids [56], their system was capable of shuttling and aggregating microplastics and other suspended matter in water samples. The magnetic material (nickel) in the coating then allowed the reclamation of the particles along with the accumulated passive waste particles. The authors also created linear chains from their swimmers to facilitate a shoveling effect for clearing illuminated surfaces of contaminant species, by effectively pushing them aside as the swimmers travelled away from UV-irradiated patches.

In addition to the catalytic and thermophoric swimmers, particles can be propelled by localized galvanic exhange reactions. Silica-based swimmers have been driven by exploiting the galvanic exchange reactions between their coating material and dissolved metal ions, in solutions at relatively low ionic strengths [63,64]. The galvanic exchange from a less noble material to a more noble material, such as, for example, from copper to platinum [63] or gold [64], exerts an electromotive force that, similarly to what occurs on the photocatalytic swimmers, induces fluid flow from the cap towards the exposed core. Thus, the particle is propelled in the direction of the reacting surface. Interestingly, after the coating material has been completely exchanged for another, the new coarse and hat-like cap could presumably be used for propulsion via different mechanisms, such as the previously described photoresponsive and catalytic propulsion techniques.

Lastly, the novel liquid metal (LM) alloys such as GaInSn are also worth highlighting, as they have seen creative use in nanoswimmer applications in recent years, in part due to their biocompatible nature. For example, the use of Ga in the core element in swimmers, instead of more traditional polymer or silica particles, has enabled their use in applications such as microwelding [65], treatment of bacterial infections [66] and swimming in alkaline environments [67]. The Ga endows the particles with bactericidal properties [66] and propulsion through self-electrophoresis or self-diffusiophoresis in NaOH containing environments depending on the coating material of the spheres (either metallic or non-metallic, respectively) [67].

### 4.2. Hybrid Swimmers

#### 4.2.1. Hybrid Swimmers Based on Capped Janus Nanospheres

A hybrid swimmer (HS) can be described as a multi-component swimmer that combines biological and artificial parts. HSs often comprise at least one biological component, the conjugation of which relies on the organic surface modification of inorganic particles. For example, noble metals such as gold and silver can be easily modified with organic thiol groups to enable attachment of the biological modifications [2]. The biological components can be used for several purposes, such as camouflaging non-biological NSs, achieving fuel-free movement and actuating directional movement taxis [68,69].

HSs can be combinations of particles, JPs and bacteria [70] and bioconjugates, such as oligonucleotides [35]. Various HSs have been proposed in the past few years and their fabrication methods are as diverse as the types of different HSs [71]. These swimmers have the potential to be used in even more intricate and sophisticated manners due to their versatility, and they have been envisioned to provide possible solutions for biomedical challenges, similarly to TDDSs.

**Figure 4:**
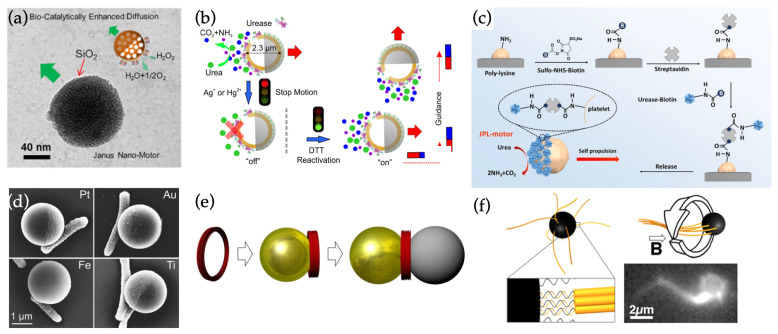
Hybrid nanoswimmers. (**a**) Silica nanosphere partially coated with catalase enables propulsion through enzymatic reactions. (**b**) Hollow mesoporous silica nanosphere partially coated with urease enzymes, which enables urea-fueled propulsion and toggling of movement by chemically inhibiting and reactivating the enzyme. (**c**) Coating a platelet cell with urease allows creation of a cell-based urea-fueled swimmer. (**d**) *E. coli* attached to capped Janus spheres provide random tumble motion to the particles, but the system can also be magnetically steered if a magnetic metal (Fe) is used for coating. (**e**) DNA origami nanoring that can be used for creating self-thermophoretic Janus-type assemblies. (**f**) Magnetic nanoswimmer with synthetic DNA origami flagella. $\vec{B}$ denotes the magnetic flux density. (**a**) reproduced from Ref. [74]. Copyright 2017 Elsevier. (**b**) reproduced from Ref. [75]. Copyright 2016 American Chemical Society. (**c**) reproduced from Ref. [48]. Copyright 2020 by American Association for the Advancement of Science. (**d**) reproduced from Ref. [70]. Copyright 2015 American Chemical Society. (**e**) reproduced from Ref. [76]. Copyright 2018 John Wiley & Sons. (**f**) reproduced from Ref. [77]. Copyright 2016 American Chemical Society.

One class of HSs are the various enzyme-powered nanomotors [72,73]. For example, catalase and urease have been harnessed for generating propulsion. Catalase enzyme-based motors operate similarly to the non-hybrid catalytic swimmers by generating hydrogen via H${}_{2}$O${}_{2}$ decomposition in a non-symmetric manner on the nanoparticle surface. In one such work, Ma et al. [74] surface-modified mesoporous silica nanospheres with carboxylic groups (-COOH) and then half-covered them with solid silica caps via electron beam evaporation (Figure 4a). After capping, the uncovered, surface-modified half could be loaded with catalase to provide propulsion at below 3 wt% H${}_{2}$O${}_{2}$ concentrations.

Similarly, urease can be employed to create swimmers based on the enzymatic decomposition of urea. Ma et al. [75] created hollow, enzyme-coated mesoporous silica spheres that were then partially coated with silica or metal (Fe/Au) with electron beam evaporation (Figure 4b). The partial coating again enabled the site-specific and directionally biased digestion of urea on the uncoated surfaces of the particles, inducing propulsion. They also showed how the swimming behavior could be toggled by chemically stifling and reactivating the enzymatic activity of the urease. Furthermore, if magnetic material was incorporated into the coating layer, magnetic steering could be used to direct the swimming of the particles. The hollow inside the spheres allowed the loading of even large particle cargo inside the swimmer, instead of merely drug molecules.

In terms of biomedical potential, the coating of non-hybrid swimmers with pieces of viral cell membrane, as has been demonstrated by coating thermophoretic CJNs with macrophage cell membranes (MPCMs), could provide swimmers with exceptional biocompatibility. In one recent work [78], the Au half-shells of Au-capped silica spheres were passivated with thiol-terminated methoxy-poly(ethylene glycol), which enabled the exposed silica faces to be fused with MPCMs. With this, the swimmers were able to retain their previous movement capabilities, but the MPCM coating could cloak them from the immune system and facilitate their targeting to cancer cells. Furthermore, propelling the particles against the cell membrane after successful targeting could assist the particles in penetrating the membrane.

Finally, it is also worth noting that the same coating strategies used for CJNs can be applied to organic structures as well. In a curious demonstration, sphere-like platelet cells were transformed into enzyme-powered nanoswimmers by incubating surface-attached platelets in a urease–biotin–Cy5 solution [48]. This allowed only the exposed cell surface to be modified with urease, essentially turning the cell into a fully biological CJN that was fueled by urea in its environment (Figure 4c).

Another category of HSs is bacteria-based swimmers [79]. As a key feature, the energy for the movement of bacteria emerges naturally from their operating environments [80]. Thus, bacteria-based hybrids aim to solve common problems of biological nanodevices, such as the need for external energy. Furthermore, the movement of these bacteria-based hybrids can be controlled remotely when integrated with a stimuli-responsive JP or by exploiting the natural stimuli responses of the bacteria components, such as chemotaxis and aerotaxis [43,68,81].

Fabrication of bacterium HSs is rather straightforward, since some bacteria, e.g., *E. coli*, may attach to surfaces simply through electrostatic forces [70]. The interactions between the bacteria and nanoparticle can be further enhanced by the addition of amino acids or other binding agents such as streptavidin, which can bind to the membrane of bacteria via biotin [81,82]. Other methods include the patterning of NSs and the blotting technique, which is based on the natural adhesion of the bacteria on contacting surfaces [80]. For movement-oriented applications such as nanoswimmers, the fabrication processes often aim to localize the bacteria to one side or part of the substrate particles [81].

Bacteria used for bio-HSs include, e.g., *E. coli* [70] and *S. marcescens* [83]. For instance, *E. coli* has been used to create drug delivery vessels in combination with polystyrene (PS) JPs. These PS particles were half-coated with a metal (Au, Pt, Fe or Ti) binding site for bacteria (Figure 4d) and the exposed PS half was used for loading a cancer drug. Bacterial adhesion to the JP was based on the natural bonding of *E. coli* to the metal coating, and the best adhesion results were attained with the Pt-capped and Fe-capped particles [70]. The attached *E. coli* provided random tumble motion to the particles. However, notably, the swimming direction of Fe-capped variants could be steered with magnetic guidance.

Recently, HSs have also demonstrated potential as biosensors, as shown in the work of Pacheco et al. [84]. They synthesized polymer-based Janus nanoswimmer sensors through the emulsion-mediated self-assembly of polycaprolactone (PCL) shells around transition metal dichalcogenides (TMDs) (MoS${}_{2}$ or WS${}_{2}$). The PCL shells were partially decorated with magnetic Fe${}_{2}$O${}_{3}$ and catalytic Pt nanoparticles during the assembly process to provide both recoverability and catalytic propulsion to the spheres. By then modifying the swimmer with a fluorescently labeled affinity peptide that quenches in the proximity of TMDs, the system could selectively produce a fluorescent signal when encountering a target bacteria endotoxin (here, from *Salmonella enterica*) that causes the peptide to detach from the swimmer.

#### 4.2.2. Hybrid Swimmers Based on Nanospheres and DNA Nanostructures

Taking the concept of multifunctional biohybrid swimmers somewhat further, also fully synthetic biomolecules have recently demonstrated their first steps in the nanoswimmer field. Instead of using the naturally occurring biological components such as bacteria and enzymes with predetermined functions, techniques including DNA origami [85,86,87] enable completely customized shape geometries as well as components with modular functionality and programmability. In general, the DNA origami technique has already been employed in creating self-thermophoretic swimmers [76,88] in conjunction with regular single-material nanospheres (Figure 4e).

Moreover, the fabrication of custom-engineered swimming tails has been demonstrated. Maier et al. [77] assembled biotinylate-modified helical DNA origami bundle flagella with different sizes and twisting and attached them on streptavidin-coated magnetic beads (Figure 4f). Upon exposure to a rotating magnetic field, the flagella formed a corkscrew-like structure behind the bead and enhanced its swimming speed. However, in their technique, the positioning and number of swimming tails was not controlled, as the entire particle was coated with them, which perhaps contributed to the rather low yields of viable swimmers (1–10% depending on tail design).

Nonetheless, very recently, their work was continued by Pauer et al. [89], when they employed CJNs for selectively localizing the attachment of artificial DNA origami flagella on magnetic nanospheres. Streptavidin modifications on a partial Au coating enabled the selective attachment of biotinylated DNA origami helix bundles on one side of the CJNs. Additionally, by using a magnetic field during the nanosphere masking and coating process, the coating and swimming tails could be aligned in respect to the magnetic easy axes of the spheres. This allowed the exact positioning of custom swimming tails on magnetic nanospheres, resulting in magnetically responsive nanoswimmers with highly adjustable swimming properties.

## 5. Conclusions and Outlook

Janus particles possess great potential as platforms for intricate modifications and thus they are promising candidates for nano- and microswimmers and drug delivery devices. Here, we have shown that these particles provide a modular basis for nanoswimmer assembly with various propulsion and steering mechanisms, as well as a myriad of potential uses (see Table 1).

With these representative cases, it becomes evident that micro- and nanoswimmers have enormous potential in, e.g., biomedical and targeted drug delivery applications [90,91]. For example, targeted drug delivery can be established in vitro using several different means of drug loading, drug release, movement control and materials. However, some of the mechanisms have attributes that impose suitability problems for large-scale production, and may exhibit traits unfit for biological systems. In addition, the diffusion and relative viscosity [92,93] experienced by swimmers in complex and crowded biological environments, such as inside cytoplasms, poses further challenges for many potential in vivo applications. To overcome these issues, several points should be resolved. For biological applications, assembly of JPs and HS should be improved in order to gain more precise, controlled and consistent products. Moreover, their fabrication processes should be standardized and become rapid, easy to scale and economical [1,94,95]. Additionally, further studies should be conducted regarding the safety of NPs in biological applications as the behavior of NPs in vivo is still quite elusive and may differ greatly from in vitro results [95].

Work in this direction is, nevertheless, constantly progressing. In general, materials such as mesoporous silica [96] have much promise as drug delivery media, and silica has indeed been used as a core material for some of the featured CJNs to achieve controlled cargo delivery. Swimmers with antibacterial materials or functions [45,66] are also a rather promising possibility with potential for biomedical applications.

In addition to non-hybrid approaches, new prospects in this field include the usage of biocomponents for resolving challenging issues regarding swimmer-based drug delivery and other biomedical uses. Some key benefits of biocomponents are their superior fit with biological systems [97], predictability of structure and behavior [98], structural variability, dynamic behavior [99] and stability under physiological conditions [100]. Overall, they present excellent selective recognition properties, which are useful in targeted drug release, gene expression control [101] and probing [35] applications.

These biological materials may result in more biocompatible devices when combined with Janus-like core particles and they have the potential for achieving immensely accurate smart tools that have foreseeable behavior and user-engineered traits. Functionalizing particles with biological modifications has shown creative uses such as bacterial sensors [84] and even cancer-targeting delivery vectors that are cloaked from the immune system [78]. By applying the coating strategies of CJNs, also the harnessing and functionalization of entire cells has been shown to be a possibility [48].

Similar applications can be fully tailored using synthetic and programmable DNA nanotechnology components [102], as has very recently been hinted at [89]. DNA origami in particular has seen increasing use as programmable drug delivery [103,104] and gene editing vectors [105] in the past few years. As such, we strongly believe that, in conjunction with CJNs and swimmers, DNA nanostructures will see growing interest when engineering custom-designed micro- and nanocarriers for TDDSs and other biomedical and non-medical applications.

## Figures and Tables

**Table 1:** Nanoswimmers classified by their type, composition, mechanism and foreseen applications.

Swimmer Type	Composition	Mechanism	Application
**Non-Hybrid Swimmers**			
Multilayer magnetic/catalytic swimmer [49]	Co/Pt multilayer on SiO${}_{2}$	H${}_{2}$O${}_{2}$ catalysis and magnetical steering	Studying swimming behavior
Magnetoresponsive swimmer [50]	CoFe${}_{2}$O${}_{4}$ and BaTiO${}_{3}$ on SiO${}_{2}$	Charge generation via interaction of magnetostrictive and piezoelectric layers	Remote controlling/triggering electrochemical reactions
Catalytic swimmer [53]	Pt on mesoporous SiO${}_{2}$	Pt catalyzed H${}_{2}$O${}_{2}$ breakdown	Drug delivery
Fuel-free photocatalytic swimmer [54]	Au coated TiO${}_{2}$	UV-light driven self-electrophoresis	Illumination controlled propulsion
Photochemically driven swimmer [56]	Cu on TiO${}_{2}$	UV light or H${}_{2}$O${}_{2}$ fuel depending on solution conditions	Studying swimming and interactions between passive and active colloids
Photochemically driven swimmers [60]	Various metals on TiO${}_{2}$	UV light and H${}_{2}$O${}_{2}$ fuel	Studying effects of coating material on swimmer propulsion
Two-directional photoresponsive swimmer [61]	Au-capped TiO${}_{2}$	Switch in reaction site (Au to TiO${}_{2}$) based on used light wavelength (UV to green)	Reversible propulsion direction
Nanocube and platelet-coated swimmers [51]	Co${}_{3}$O${}_{2}$ nanocubes or platelets on TiO${}_{2}$	UV-light-driven photocatalytic/self-electrophoretic propulsion	Studying effects of coating morphology on swimming
Photochemically driven polymer-cored swimmer [62]	AgCl on PMMA	UV-and-visible-light-driven decomposition of AgCl to Ag	Studying ionic self-diffusiophoresis
Photothermal swimmer [52]	Au on SiO${}_{2}$	Light-induced thermal gradient between cap and uncoated half	Fuel-free light-controlled propulsion
Magnetothermal swimmer [24]	Permalloy on SiO${}_{2}$	Asymmetric heating of particle with AC magnetic field, steering with DC magnetic field	Magnetic steering and propulsion
Multilayered antibacterial swimmer [45]	Ag on Au on Fe on Mg	Mg-based propulsion, magnetic guidance and collection, bacterial adhesion and Ag release	Killing and collecting bacteria in water
Photocatalytic magnetic swimmer [57]	Ni and Au on TiO${}_{2}$	UV-driven propulsion, magnetic reclaiming	Herding, aggregating and collecting passive colloidal species in solution
Galvanic exchange swimmers [63,64]	Metal coating on SiO${}_{2}$	Galvanic exchange of partial coating with more noble metal in solution induces an electromotive force	Capping synthesis and material exchange, switching of propulsion mechanism
Liquid metal alloy swimmers [65,66,67]	Capped liquid metal core	Self-diffusiophoresis (non-metallic) or self-electrophoresis (metal) depending on cap material	Propulsion in alkaline environments, biocompatible and bactericidal swimmers, microwelding
**Hybrid Swimmers**			
Catalase swimmer [74]	SiO${}_{2}$-capped catalase-modified mesoporous SiO${}_{2}$	H${}_{2}$O${}_{2}$ breakdown on catalase-coated side	Biocatalytic propulsion, drug delivery
Hollow-cored urease swimmer [75]	Urease-coated mesoporous SiO${}_{2}$ with SiO${}_{2}$, Fe or Au capping	Propulsion via urea decomposition, magnetic steering (with Fe cap), cargo space within particle	Controlled enzymatic swimming, delivery of large-particle cargo
Cell-membrane-coated swimmer [78]	Au-capped SiO${}_{2}$ with macrophage cell membrane on exposed SiO${}_{2}$	Thermophoretic propulsion, cloaking and cell-specific targeting due to cell membrane coating	Immunological cloaking, cancer cell targeting, assisted cell membrane penetration
Capped platelet cells [48]	Urease-capped platelet cells	Urea-fueled propulsion, retained cell functionality	Harnessing of cells as nanoswimmers
*E. coli*-based swimmer [70]	*E. coli* attached to metal-capped polystyrene	Random tumble motion of *E. coli*, magnetic steering (with Fe coatings)	Fuel-free random or directed propulsion, drug delivery
Biosensor swimmer [84]	MoS${}_{2}$ or WS${}_{2}$ inside polymer shell partially coated with metals and fluorescent affinity peptide	Catalytic propulsion, magnetic steering and collecting, peptide release and light emission upon encountering target endotoxin	On/off-type species-selective biosensor for bacteria detection
**DNA-Based Swimmers**			
Thermophoretic DNA origami swimmers [76,88]	Custom DNA origami structures on Au particles	Propulsion via asymmetric heating during illumination	Platform for thermophoretic swimmers with adjustable behavior
Magnetic swimmers with DNA origami tails [77,89]	DNA origami flagella conjugated to Au-capped magnetic beads	Flagella-mediated propulsion during magnetic rotation of beads	Custom engineering of movement behavior
